# The relationships between school children's wellbeing, socio-economic disadvantage and after-school activities: a cross-sectional study

**DOI:** 10.1186/s12887-022-03322-1

**Published:** 2022-05-21

**Authors:** Eliza Kennewell, Rachel G. Curtis, Carol Maher, Samuel Luddy, Rosa Virgara

**Affiliations:** 1 Alliance for Research in Exercise, Nutrition and Activity, UniSA Allied Health and Human Performance, University of South Australia, GPO Box 2471, Adelaide, South Australia 5001 Australia; 2 System Performance Division, Government of South Australia Department for Education, 31 Flinders St Adelaide, Adelaide, South Australia 5001 Australia

**Keywords:** After-school, Activities, Wellbeing, Children

## Abstract

**Background:**

Lower socioeconomic status is associated with poorer wellbeing among children. Identifying how children participate in after-school activities and how after-school activities are associated with wellbeing may inform interventions to improve wellbeing among children from low socioeconomic backgrounds. This study explored whether children’s after-school activities varied by socioeconomic status and examined the associations between after-school activities and wellbeing in low socioeconomic status children.

**Methods:**

This study analysed cross-sectional data from 61,759 school students in years 4 to 9 who completed the 2018 South Australian Wellbeing and Engagement Collection. Students reported the number of days per week they participated in 12 activities (after-school care, homework, music lessons or practice, youth organisations, sports, television, videogames, social media, reading, chores, arts and crafts, and socialising with friends) during the after-school period (3-6 pm) and their wellbeing (happiness, sadness, worry, engagement, perseverance, optimism, emotion regulation, and life satisfaction). Socioeconomic status was measured by parents' highest education level obtained from school enrolment data. Linear multilevel models were used to examine whether frequency of after-school activities varied by socioeconomic status. Multilevel ordered logit models were used to analyse the association between after-school activities and wellbeing amongst participants in the low socioeconomic status category.

**Results:**

After-school activities differed according to socioeconomic status; high socioeconomic status children did more frequent sport, homework, and reading and low socioeconomic status children did more frequent screen-based activities (TV, videogames and social media). Among children from low socioeconomic status backgrounds, higher wellbeing was associated most consistently with more frequent sports participation, homework, reading and spending time with friends and less frequent videogames, social media and after-school care.

**Conclusions:**

Children's wellbeing is positively associated with socioeconomic status. Amongst children from disadvantaged backgrounds, participating in sport, spending time with friends and getting less screen time may be protective for wellbeing. The results suggest that programming targeted at increasing sports participation and reducing screen time amongst children from low socioeconomic status backgrounds may support their wellbeing.

**Supplementary Information:**

The online version contains supplementary material available at 10.1186/s12887-022-03322-1.

## Introduction

The World Health Organization defines health as "a state of complete physical, mental and social wellbeing and not merely the absence of disease or infirmity" [1]. Similarly, mental health is not merely the absence of mental health disorders, but “a state of well-being in which an individual realizes his or her own abilities, can cope with the normal stresses of life, can work productively and is able to make a contribution to his or her community” [2]. Mental or subjective wellbeing, hereafter referred to as simply “wellbeing”, is a multifaceted construct typically considered to comprise emotional states such as happiness and sadness and cognitive evaluations such as life satisfaction [3]. According to the Australian Institute of Health and Welfare [4], children aged between 5 and 12 years are at an important stage for health and wellbeing as the transition into full time school brings about challenges and risks. Since the start of the twenty-first century, wellbeing has been increasing in importance in political debate and policy. In particular, concerns about childhood wellbeing are prominent [5].

International research shows that mental health and wellbeing is poorer in children from low socioeconomic status (SES) backgrounds in countries such as Denmark [6], Norway [7], Iceland [8], Hong Kong [9], and the United States [10]. Additionally, an Australian study found that children from low SES backgrounds had poorer psychological functioning and were more likely to have their activities impacted by poor health or emotional/behavioural problems [11]. Socioeconomically disadvantaged children may be two to three times more likely to develop mental health problems [12]. A range of factors may contribute to poorer wellbeing among children from low SES backgrounds including family stress, poor parental mental health and parenting behaviours, and resource limitations (e.g., education and housing) [13]. Interventions to improve wellbeing in low SES children are needed.

One potential target for intervention is after-school activities. Participation in after-school activities appears to differ between children from different SES backgrounds and be associated with wellbeing. Participation in organised after-school activities (such as sports, dancing, and clubs) often have an associated cost, which may be prohibitive for low income families. Research has shown that higher household income is associated with more participation in organised activities such as music lessons, art classes, and club-based activities such as boy scouts among 8 to 9 year-olds [14]. Similarly, adolescents from high-income families spend more time playing musical instruments and playing sport [15]. Participation in organised after-school activities provides children an opportunity to develop social and cognitive skills [16]. Adolescents who participate in organised after-school activities have shown lower depressed mood than adolescents who do not [17] while children who participate in organised after-school activities display more adaptive behaviour than children who do not [16]. Participation in greater number of organised after-school activities has also been linked to better psychological resilience [18]. Specific activities may play a role. For example, regular participation in physical activity has been shown to enhance the wellbeing of children [19, 20].

Non-organised after-school activities may also contribute to wellbeing. Children in high-income families spend more time reading and doing homework [15]. Conversely, children in low-income families spend more time watching television and playing videogames [15]. Higher levels of screen time have been shown to be associated with poorer wellbeing in children [21, 22].

If children from low SES backgrounds have different patterns of participation in after-school activities than children from high SES backgrounds, and if participation in after-school activities influences wellbeing of children from low SES backgrounds, interventions to alter after-school activity patterns could potentially improve their wellbeing. For example, a 2015 study showed that 54% of the after-school period was spent sedentary [23] but children can obtain up to 30–50% of their daily recommended PA in the after-school period alone [23, 24]. Activities offered in the after-school period are an opportunity to increase children’s physical activity [25] and may improve wellbeing. Identifying how after-school activities are associated with wellbeing specifically in children from low SES backgrounds is the first step in considering a wellbeing intervention targeting after-school activities. This study therefore aimed to 1) examine whether Australian children’s after-school activities vary on the basis of SES and 2) examine the association between frequency of after-school activities and wellbeing among Australian children from low SES backgrounds.

## Methods

### Research design

A cross-sectional analysis was conducted on data from the 2018 South Australian Well-being and Engagement Collection (referred to as “census” from here-on in this paper), collected by the South Australian Department for Education (SA Dept Ed) [26]. The census, which began in 2013, is an annual self-report survey that examines wellbeing, developmental health, school experiences and engagement during school and in the after-school period among government school students in years 4 to 9 (9 to 15 years of age). Since its introduction, the participation rate in the census has increased, reaching 93% participation of government schools in 2018. Twenty nine percent of Catholic and 18% of Independent schools also participated [26]. The SA Dept Ed reviewed and approved the use of the census data for further analysis (approval no. 2019–7,313,841). This secondary data analysis was exempt from ethics approval from the University of South Australia’s Human Research Ethics Committee (application no. 202625).

### Participants and design

The census was an online survey that was administered during school hours between July and August 2018. A small number of schools administered a paper-based survey. Students completed the survey in class individually under the supervision of either a classroom teacher, principal, or other adult staff member. To be eligible, participants must have been a school student in years 4–9 in South Australia. Parental opt-out consent procedure was used which maximised the participation rate and therefore the representativeness of the data. Students were also given the option of withdrawing from the survey as a whole or of choosing not to answer any specific questions. School staff administering the survey advised students of their right not to participate before the survey commenced. In addition, the survey instructions repeated this message. 61,759 participants with sufficient available data were included in the dataset for analysis.

### Variables

#### Social and emotional wellbeing

Happiness, engagement, and perseverance were each measured with three items from the EPOCH Measure of Adolescent Well-Being [27]. Sadness and optimism were each measured with three items from the Middle Years Development Instrument [28]. Emotion regulation was measured with three items from the Emotion Regulation Questionnaire for Children and Adolescents [29]. Life satisfaction was measured with five items from the Satisfaction with Life Scale for Children [30]. Worry was measured with 4 items developed by the SA Dept Ed and the Telethon Kids Institute. Research has shown good internal consistency and test–retest reliability for these scales (happiness α = 0.86, 3-week *r* = 0.71, engagement α = 0.74, 3-week *r* = 0.63, perseverance α = 0.80, 3-week *r* = 0.69 [27]; sadness α = 0.70, optimism α = 0.66 [28]; emotion regulation α = 0.82, 2-month *r* = 0.67 [29, 31]; life satisfaction α = 0.86 [30]). The scales have also shown good construct validity. For example, the EPOCH happiness scale is positively associated with physical vitality (*r* = 0.58) and meaning/purpose (*r* = 0.55), and negatively associated with depressive symptoms (*r* = -0.53) [27], while the Middle Years Development Instrument optimism scale is associated with life satisfaction (*r* = 0.57) [28]. For all items, participants responded on a 5-point Likert scale from 1 = strongly disagree/almost never/not at all like me to 5 = strongly agree/almost always/very much like me. Items on each scale were averaged to create a scale score from 1 to 5 and scale scores were categorised as “low” (students scored < 3), “medium” (students scored ≥ 3 or < 4), or “high” (students scored ≥ 4) [32].

#### After-school activities

After-school activities were assessed using items created as part of the Middle Years Development Instrument [28]. Participants were asked to report the number of days (never, once a week, twice a week, 3 times a week, 4 times a week, or 5 times a week) they participated in each organised activity (after-school care, homework or educational lessons, music lessons or practice, youth organisations, organised individual or team sports), and other activities (television, videogames, social media, reading, chores, arts and crafts, “hanging out” with friends) during the after-school period (3-6 pm).

#### Demographic variables

Key demographic data were obtained from school enrolment information held by the SA Dept Ed. These included year level, gender, and SES. For the purpose of this study, SES was based on the student’s parent or caregiver with the highest education level, with low SES defined as completed year 12 of high school or less, medium SES as having completed Certificate/Diploma/Advanced diploma education, and high SES as having completed a Bachelor degree or higher.

### Statistical methods

Descriptive statistics (means, standard deviations, percentages) were used to describe demographic variables and frequency of after-school activities. Multilevel ordered logit models with mean–variance adaptive Gauss–Hermite quadrature were used to confirm the expected association between SES and wellbeing using the full sample. Analyses controlled for school year level and gender and included a random effect for school to account for the structure of the data (students nested within schools). Separate models were conducted for each wellbeing variable. Multilevel ordered logit models were used due to the ordinal nature of the wellbeing variables. Linear multilevel models with maximum likelihood estimation were used to examine whether the frequency of after-school activities varied by SES, controlling for school year level and gender and including a random effect for school. Separate models were conducted for each activity. Finally, multilevel ordered logit models with mean–variance adaptive Gauss–Hermite quadrature were used to analyse the association between after-school activities and wellbeing using only participants in the low SES category including a random effect for school. Separate models were conducted for each of the wellbeing variables. All models used listwise deletion for missing data on the independent variables. Analyses were conducted in Stata 13 with a *p*-value of 0.05 used to denote statistical significance (StataCorp. Stata Statistical Software. College Station, TX: StataCorp LP; 2013).

## Results

Approximately 10,000 students per year level from Year 4 through to Year 9 participated; most were from families belonging to the medium SES category (46.2%) as shown in Table 1. Amongst students who participated, gender was evenly distributed by school year level.

**Table 1:** Participant demographic characteristics

Variable	n (%)		
	Male	Female	All
*Year level (n* = *61,759)*			
Year 4	5584 (17.7)	5466 (18.0)	11,050 (17.9)
Year 5	5765 (18.3)	5675 (18.7)	11,440 (18.5)
Year 6	5691 (18.1)	5542 (18.3)	11,233 (18.2)
Year 7	5318 (16.9)	5089 (16.8)	10,407 (16.9)
Year 8	4620 (14.7)	4478 (14.8)	9098 (14.7)
Year 9	4494 (14.3)	4037 (13.3)	8531 (13.8)
Total	31,472 (51.0)	30,287 (49.0)	61,759 (100)
*SES (n* = *56,958)*			
Low	6530 (22.5)	6483 (23.2)	13,013 (22.8)
Medium	13,409 (46.3)	12,880 (46.0)	26,289 (46.2)
High	9031 (31.2)	8625 (30.8)	17,656 (31)

*SES* Socioeconomic status

Table 2 shows results from multilevel ordered logit models examining the association between SES and wellbeing. For each model, the likelihood ratio chi-square test showed there was enough variability between schools to use a multilevel model (happiness χ2(1) = 576.54, *p* < 0.001; sadness χ2(1) = 421.64, *p* < 0.001; worry χ2(1) = 381.96, *p* < 0.001; emotion regulation χ2(1) = 296.92, *p* < 0.001; life satisfaction χ2(1) = 373.12, *p* < 0.001; engagement χ2(1) = 366.37, *p* < 0.001; optimism χ2(1) = 443.95, *p* < 0.001; perseverance χ2(1) = 579.29, *p* < 0.001). Compared to students in the low SES category, students in the high SES category were 62% more likely to score higher on perseverance (OR 1.62, 95% CI 1.55, 1.70), 34% more likely to score higher on happiness (OR 1.34, 95% CI 1.28, 1.42), 29% more likely to score higher on optimism (OR 1.29, 95% CI 1.28, 1.42), and 23% more likely to score higher on life satisfaction (OR 1.23, 95% CI 1.17, 1.29). Compared to students in the low SES category, students in the high SES category were also 6% more likely to score higher on emotion regulation (OR 1.06, 95% CI 1.01, 1.11) and engagement (OR 1.06 95% CI 1.02, 1.12). Students in the high SES category were 30% less likely to score higher on worry (OR 0.70, 95% CI 0.67, 0.73) and 9% more likely to score high on sadness (OR 1.09, 95% CI 1.07, 1.10) than children in the low SES category.

**Table 2:** Odds ratios [and 95% confidence intervals] from multilevel ordered logit models examining the association between socioeconomic status and wellbeing

	Happiness OR [95%CI]	Sadness OR [95%CI]	Worry OR [95%CI]	Emotion regulation OR [95%CI]	Life satisfaction OR [95%CI]	Engagement OR [95%CI]	Optimism OR [95%CI]	Perseverance OR [95%CI]
Girls^a^	0.92 [0.89, 0.96]***	1.44 [1.39, 1.49]***	1.59 [1.54, 1.64]***	0.90 [0.87, 0.93]***	0.81 [0.78, 0.83]***	0.90 [0.87, 0.93]***	0.87 [0.84, 0.90]***	1.40 [1.35, 1.44]***
School year level	0.86 [0.84, 0.87]***	1.09 [1.07, 1.10]***	1.07 [1.06, 1.08]***	0.83 [0.82, 0.84]***	0.82 [0.81, 0.83]***	0.87 [0.86, 0.88]***	0.82 [0.81, 0.83]***	0.90 [0.89, 0.91]***
*SES*								
Medium SES^b^	1.13 [1.09, 1.18]***	0.88 [0.85, 0.92]***	0.90 [0.86, 0.93]***	0.96 [0.92, 1.00]*	1.08 [1.04, 1.13]***	0.99 [0.95, 1.03]	1.07 [1.02, 1.11]**	1.20 [1.15, 1.25]***
High SES^b^	1.34 [1.28, 1.42]***	1.09 [1.07, 1.10]***	0.70 [0.67, 0.73]***	1.06 [1.01, 1.11]*	1.23 [1.17, 1.29]***	1.06 [1.02, 1.12]**	1.29 [1.23, 1.36]***	1.62 [1.55, 1.70]***

*SES* Socioeconomic status

^a^reference group is boys

^b^reference group is low SES

^*^p < .05, ^**^p < .01, ^***^p < .001

Table 3 shows the average days per week students participated in each after-school activity, split by SES. Amongst all students, the most common activities were watching TV (*M* = 3.9, *SD* = 1.5), spending time on social media (*M* = 2.9, *SD* = 2.2), and participating in household chores (*M* = 3.2, *SD* = 1.7). The least common activities were attending youth organisations (e.g. Scouts, Girl Guides, Boys and Girls Club; *M* = 0.4, *SD* = 1.1), after-school care (*M* = 0.5, *SD* = 1.2) and music (*M* = 0.9, *SD* = 1.6). Students in the high SES category participated in sports, homework, reading and playing music on more days per week than students in medium and low SES categories. Students from low SES backgrounds spent more days per week socialising with friends, watching TV, playing videogames and using social media than students in the high SES category. The activities that varied the least across all SES categories were after-school care, chores, youth organisations and arts and crafts.

**Table 3:** Frequency of after-school activities split by SES

Activity	Days per week *M* (*SD*)			
	Low SES	Medium SES	High SES	All
After-school care	0.5 (1.3)^ab^	0.5 (1.2)^a^	0.5 (1.2)^b^	0.5 (1.2)
Sports	1.8 (1.8)	2.0 (1.7)	2.2 (1.7)	2.0 (1.7)
Homework	2.3 (1.9)	2.4 (1.9)	2.7 (1.9)	2.5 (1.9)
TV	4.0 (1.5)^a^	4.0 (1.5)^a^	3.7 (1.5)	3.9 (1.5)
Videogames	2.9 (2.0)	2.7 (2.0)	2.3 (1.97)	2.6 (2.0)
Social media	3.1 (2.1)	3.1 (2.1)	2.5 (2.2)	2.9 (2.2)
Read	2.0 (2.0)	2.1 (2.0)	2.8 (2.0)	2.3 (2.0)
Chores	3.0 (1.8)	3.2 (1.8)	3.3 (1.7)	3.2 (1.7)
Music	0.8 (1.5)^a^	0.8 (1.5)^a^	1.1 (1.7)	0.9 (1.6)
Friends	2.6 (1.9)^a^	2.6 (1.9)^a^	2.3 (1.9)	2.5 (1.9)
Arts and crafts	1.5 (1.6)^a^	1.4 (1.7)^b^	1.5 (1.7)^ab^	1.5 (1.7)
Youth Organisations	0.5 (1.2)	0.4 (1.1)	0.3 (0.9)	0.4 (1.1)

*SES* Socioeconomic status

^ab^For SES groups, means in a row without a common superscript letter differ (*p* < 0.05), according to multilevel modelling with Tukey post-hoc tests, controlling for school year level and gender

**Fig. 1:**
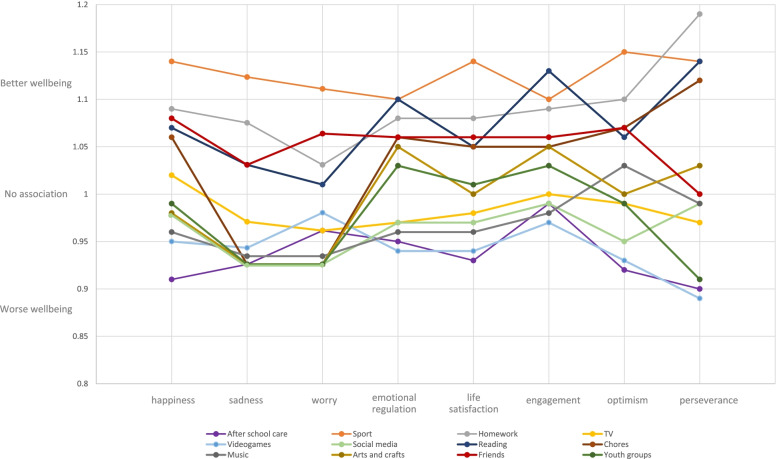
Odds ratios comparing after-school activities and wellbeing among students from a low socioeconomic background. Note: sadness and worry have been reversed scored

Figure 1 shows odds ratios from multilevel ordered logit models examining the association between after-school activities and wellbeing among students from a low SES background (*n* = 13,013; full results can be found in Supplementary Table 1, Additional file 1). For each model, the likelihood ratio chi-square test showed there was enough variability between schools to use a multilevel model (happiness χ2(1) = 34.91, *p* < 0.001; sadness χ2(1) = 10.21, *p* < 0.001; worry χ2(1) = 14.57, *p* < 0.001; emotion regulation χ2(1) = 12.70, *p* < 0.001; life satisfaction χ2(1) = 28.36, *p* < 0.001; engagement χ2(1) = 35.45, *p* < 0.001; optimism χ2(1) = 8.76, *p* < 0.001; perseverance χ2(1) = 17.33, *p* < 0.001). Most notably, low SES students who played sport more frequently had consistently higher scores across the range of wellbeing metrics, that is they were 15% more likely to score higher on optimism, 14% higher scores on happiness, life satisfaction and perseverance; 10% higher scores on emotional regulation and engagement.. Smilarly, doing more homework and spending time with friends were almost consistently associated with better wellbeing. ( i.e. 8–9% more likely to score higher on happiness, 6–8% on emotional regulation & life satisfaction, 6–9% on engagement, 7–10% on optimism and 19% for perseverance). Conversely, attending after-school care, playing video games, and using social media were almost consistently associated with poorer wellbeing (i.e. 5–9% less likely to score higher on happiness, 3–5% on emotional regulation, 2–7% on life satisfaction, 1–2% on engagement, 7–8% on optimism and 10–11% for perseverance).

## Discussion

This study examined how children’s after-school activities varied on the basis of SES and the association between after-school activities and wellbeing in low SES children. As expected in line with previous literature, children from low SES backgrounds generally had poorer wellbeing than children from high SES backgrounds. Compared with children from high SES backgrounds, children from low SES backgrounds participated in less frequent after-school sport, homework, reading and chores, and participated in more frequent after-school screen time (TV, videogames, and social media) and youth organisations. Amongst the low SES children, participation in sport, homework, reading and spending time with friends were associated with better wellbeing outcomes.

This study found that the most frequent after-school activity was watching TV. Other frequently-reported activities were other sedentary activities (e.g. social media and videogames), and chores. This is consistent with previous research that demonstrated most Australian children exceed sedentary behaviour guidelines [33]. After-school activity patterns varied based on SES. Children from high SES backgrounds participated in sports, reading, homework and playing musical instruments more frequently children from low SES backgrounds. Children from low SES families spent time with friends, watched TV, played videogames, and used social media more frequently than children from high SES backgrounds. This could be because children from low SES backgrounds have less opportunity to participate in organised activities. Humbert et al. [34] ran focus groups with children and adolescents, and identified that low SES children face a range of barriers to physical activity participation, namely proximity/transport to activities, costs associated with organised activities, lack of equipment/facilities and neighbourhood safety concerns. One study showed children from a high-income household were more involved in club sports, which in turn allowed opportunities to be more active [35]. Some studies have shown that higher SES is related to higher physical activity levels [36, 37]. However, this is contradicted by evidence suggesting that SES does not affect overall physical activity levels in children as low SES children are more likely to participate less in formal sports activities but more in informal sports and active play [38].

Amongst low SES children, more frequent participation in sport was associated with better wellbeing outcomes. This confirms previous research that physical activity enhances the health and wellbeing of children [19, 20]. Additionally, participation in homework, reading and spending time with friends were associated with better wellbeing outcomes among low SES children. Research has indicated that cognitive competence (e.g., reading, writing, and critical thinking skills) and social-emotional competence (e.g., collaboration skills, motivation, and study skills) are important predictors of academic achievement and wellbeing [39, 40]. Socialising with friends has also been found in previous research to be central to wellbeing of children [41]. The findings that sport, homework, reading and spending time with friends are associated with wellbeing specifically in children from low SES backgrounds suggests that increasing these after-school activities may be beneficial for the wellbeing of socio-economically disadvantaged children.

Amongst low SES children, more frequent videogames and social media were associated with poorer wellbeing outcomes. Exceeding sedentary behaviour guidelines is associated with poorer health and wellbeing in children [23, 42]. A study conducted in the United States showed a drop in adolescents’ wellbeing from 2012–2016, which was possibly due to the increase in time spent on electronic communication with the adoption of smartphone technology [43]. Reducing these screen-time activities may be beneficial for the wellbeing of low SES children.

### Strengths and limitations

The large sample size and high participation rate of children among government schools was a major strength of this study, improving confidence that the results are representative of children attending government schools across the entire state (the largest school sector in South Australia). However, a potential source of bias was the lack of wide-spread participation from private independent and Catholic schools. Certain groups may have also been underrepresented in the data. For example, students who were unable to read or engage in the census and students who were absent from school on the day the census was completed. Additionally, due to the large sample size, many statistically significant associations were detected which may reflect negligible differences [44]. A further important limitation of the study was the cross-sectional design, which does not provide the opportunity to assess causal relationships.

### Implications

Study results highlight that SES is associated with children’s wellbeing, with children from low SES backgrounds reporting poorer wellbeing. However, results also highlight that amongst low SES children, engagement in certain types of activities is associated with higher wellbeing. In particular, more frequent engagement in organised sports, homework, reading and spending time with friends was associated with higher wellbeing. Low SES students who participated in these after-school activities had similar wellbeing to high SES students in some wellbeing categories. For example, the percentage of low SES students with high happiness was 45% for those who never played sport but 59% for those who ever played sport (once per week or more) – similar to 65% of high SES students with high happiness. Similarly, the percentage of low SES students with high emotion regulation was 32% for those who never played sport but 44% for those who ever played sport – similar to 42% of high SES students with high emotion regulation. Findings were similar for reading. For example, the percentage of low SES students with high satisfaction was 35% for those who never read but 46% for those who ever read – similar to 49% of high SES students with high satisfaction. Similarly, the percentage of low SES students with high emotion regulation was 30% for those who never read but 46% for those who ever read – more than 42% of high SES students with high emotion regulation. Although this study cannot determine causation (i.e., that the activities influenced wellbeing), findings may suggest that programs promoting these activities amongst children from low SES backgrounds are needed. For example, results provide support for the continued funding of government programmes such as School Sports Vouchers (a Government program providing South Australian primary-school aged children an annual voucher for up to $100 for sports, swimming lessons or dance fees) to encourage children from lower SES to participate in physical activity opportunities.

## Conclusion

This study found that the most frequent after-school activities among children from all SES backgrounds were screen-based activities, such as TV, videogames, and social media, and chores. Students from low SES backgrounds participated in sports, homework, reading and playing music on fewer days per week than students from high SES backgrounds, and spent time with friends, watched TV, played videogames, and used social media on more days per week than students from high SES backgrounds. Amongst children from low SES backgrounds, screen-based activities were negatively associated with nearly all wellbeing constructs whilst sport, "hanging out" with friends and doing homework were positively associated with wellbeing outcomes. These results suggest that children from low SES backgrounds may benefit from increased physical activity, socialisation and homework after-school and less time engaged in screen-based activities. Future research should focus on understanding the causation of the relationship between these activities and wellbeing.

## Supplementary Information

**Additional file 1: Supplementary Table 1.** Odds ratios [and 95% confidence intervals] examining after-school activities and wellbeing among students from a low socioeconomic background.

## Data Availability

SA Dept Ed policies support the release of data for research purposes, subject to the approval of a formal request to the department through its research application process. Queries can be directed to education.researchunit@sa.gov.au. Data tables used in this study are available from the authors upon reasonable request and with permission of the SA Dept Ed.

## Abbreviations

SES: Socio-economic status
The SA Dept Ed: South Australian Department for Education
OR: Odds Ratio
