# Supplementary material for: The relationships between school children's wellbeing, socio-economic disadvantage and after-school activities: a cross-sectional study
Source: BMC Pediatr. 2022 May 21;22:297. doi: 10.1186/s12887-022-03322-1 (PMC9123778; doi:10.1186/s12887-022-03322-1)
Supplement: Supplementary file 1 — Additional file 1: Supplementary Table 1. Odds ratios [and 95% confidence intervals] examining after-school activities and wellbeing among students from a low socioeconomic background. [file 12887_2022_3322_MOESM1_ESM.docx]

Supplementary Table 1. Odds ratios [and 95% confidence intervals] examining after-school activities and wellbeing among students from a low socioeconomic background.

|  | Happiness  OR [95%CI] | Sadness  OR [95%CI] | Worry  OR [95%CI] | Emotion regulation  OR [95%CI] | Life satisfaction  OR [95%CI] | Engagement  OR [95%CI] | Optimism  OR [95%CI] | Perseverance  OR [95%CI] |
| --- | --- | --- | --- | --- | --- | --- | --- | --- |
| After-school care | 0.91 [0.87, 0.94]*** | 1.08 [1.04, 1.12]*** | 1.04 [1.00, 1.08]* | 0.95 [0.92, 0.98]* | 0.93 [0.90, 0.97]*** | 0.99 [0.95, 1.02] | 0.92 [0.88, 0.95]*** | 0.90 [0.87, 0.93]*** |
| Sport | 1.14 [1.11, 1.17]*** | 0.89 [0.87, 0.91]*** | 0.90 [0.88, 0.93]*** | 1.10 [1.08, 1.13]*** | 1.14 [1.12, 1.17]*** | 1.10 [1.08, 1.13]*** | 1.15 [1.12, 1.17]*** | 1.14 [1.11, 1.17]*** |
| Homework | 1.09 [1.06, 1.11]*** | 0.93 [0.91, 0.95]*** | 0.97 [0.95, 0.99]** | 1.08 [1.05, 1.10]*** | 1.08 [1.05, 1.10]*** | 1.09 [1.07, 1.11]*** | 1.10 [1.08, 1.12]*** | 1.19 [1.17, 1.22]*** |
| TV | 1.02 [0.99, 1.04] | 1.03 [1.00, 1.05] | 1.04 [1.01, 1.07]** | 0.97 [0.94, 1.00]* | 0.98 [0.96, 1.01] | 1.00 [0.98, 1.03] | 0.99 [0.91, 0.95]*** | 0.97 [0.94, 0.99]* |
| Videogames | 0.95 [0.93, 0.97]*** | 1.06 [1.03, 1.08]*** | 1.02 [1.00, 1.05]* | 0.94 [0.92, 0.96]** | 0.94 [0.92, 0.96]*** | 0.97 [0.95, 0.99]** | 0.93 [0.91, 0.95]*** | 0.89 [0.87, 0.91]*** |
| Social media | 0.98 [0.96, 1.00] | 1.04 [1.02, 1.07]*** | 1.07 [1.04, 1.10]*** | 0.97 [0.95, 0.99]*** | 0.97 [0.95, 0.99]** | 0.99 [0.97, 1.01] | 0.95 [0.93, 0.97]*** | 0.99 [0.96, 1.01] |
| Reading | 1.07 [1.04, 1.09]*** | 0.97 [0.95, 1.00]* | 0.99 [0.97, 1.01] | 1.10 [1.08, 1.13]*** | 1.05 [1.02, 1.08]*** | 1.13 [1.12, 1.16]*** | 1.06 [1.04, 1.09]*** | 1.14 [1.12, 1.17]*** |
| Chores | 1.06 [1.03, 1.08]*** | 0.98 [0.96, 1.01] | 1.01 [0.99, 1.03] | 1.06 [1.04, 1.09]*** | 1.05 [1.03, 1.08]*** | 1.05 [1.02, 1.07]*** | 1.07 [1.05, 1.09]*** | 1.12 [1.10, 1.15]*** |
| Music | 0.96 [0.93, 0.99]** | 1.07 [1.04, 1.10]*** | 1.07 [1.04, 1.10]*** | 0.96 [0.93, 0.98]** | 0.96 [0.93, 0.99]* | 0.98 [0.95, 1.01] | 1.03 [1.00, 1.06]*** | 0.99 [0.96, 1.02] |
| Arts and crafts | 0.98 [0.96, 1.01] | 1.03 [1.00, 1.06]* | 1.03 [1.00, 1.05] | 1.05 [1.02, 1.08]*** | 1.00 [0.97, 1.03] | 1.05 [1.03, 1.08]*** | 1.00 [1.00, 1.06] | 1.03 [1.00, 1.06]* |
| Friends | 1.08 [1.06, 1.10]*** | 0.97 [0.95, 0.99]** | 0.94 [0.92, 0.96]*** | 1.06 [1.04, 1.08]*** | 1.06 [1.04,1.08]*** | 1.06 [1.04, 1.08]*** | 1.07 [1.06, 1.10]*** | 1.00 [0.97, 1.02] |
| Youth organisations | 0.99 [0.95, 1.03] | 1.06 [1.02, 1.11]** | 1.09 [1.05, 1.13]*** | 1.03 [0.99, 1.07]*** | 1.01 [1.00, 1.08] | 1.03 [0.99, 1.07] | 0.99 [0.95, 1.03] | 0.91 [0.87, 0.95]*** |

Note: Each wellbeing outcome was run as a separate model. Analyses controlled for gender and school year level.

*p < .05; **p < .01; ***p < .001
